# Festschrift in honor of Dr. Jeffrey Hymes

**DOI:** 10.3389/fneph.2025.1585713

**Published:** 2025-05-28

**Authors:** Terry Ketchersid, Dinesh K. Chatoth, Robert J. Kossmann, Chance Mysayphonh, Peter Kotanko, Franklin W. Maddux

**Affiliations:** ^1^ Interwell Health, Waltham, MA, United States; ^2^ Fresenius Medical Care, Waltham, MA, United States; ^3^ Renal Research Institute, New York, NY, United States; ^4^ Icahn School of Medicine at Mount Sinai, New York, NY, United States

**Keywords:** hemodialysis, hepatitis C - chronic, anemia, hyperparathyroidism, COVID-19

## Abstract

This Festschrift in honor of Dr. Jeffrey Hymes, a distinguished leader in nephrology and a pioneer in the field of dialysis care. Dr. Hymes’ career has been marked by his unwavering commitment to improving patient outcomes through innovative approaches and data-driven insights. His contributions have not only advanced the practice of nephrology but have also had a profound impact on the lives of countless patients.

## Introduction

“A life is not Important except in the impact it has on other lives” (Jackie Robinson)

It is with great admiration and respect that we present this Festschrift in honor of Dr. Jeffrey Hymes, a distinguished leader in nephrology and a pioneer in the field of dialysis care. Dr. Hymes’ career has been marked by his unwavering commitment to improving patient outcomes through innovative approaches and data-driven insights. His contributions have not only advanced the practice of nephrology but have also had a profound impact on the lives of countless patients. Held on October 25, 2024, during the American Society of Nephrology meetings in San Diego, California, several short addresses were delivered in Jeff’s honor to celebrate his contributions to the field and his leadership within the nephrology and kidney care community. This Festschrift is a result of that event.

Dr. Hymes’ journey with Fresenius Medical Care has been one of remarkable achievements. Under his leadership, the organization has harnessed the power of data to drive clinical excellence and improve patient care. From the early adoption of electronic health records to the development of sophisticated clinical algorithms, Dr. Hymes has been at the forefront of leveraging technology to enhance healthcare delivery.

Dr. Hymes’ dedication to patient safety is exemplified by his role in the careful rollout and monitoring of long-acting erythropoiesis-stimulating agents (ESAs). His meticulous approach to data analysis and patient care has led to significant advancements in the management of anemia in dialysis patients, ensuring better outcomes and improved quality of life.

A notable accomplishment is his work in the management of chronic kidney disease-mineral and bone disorders (CKD-MBD). His leadership in the utilization of calcimimetics has set a benchmark for effective and fiscally responsible treatment strategies. Similarly, his efforts in the eradication of hepatitis C among patients with end-stage kidney disease (ESKD) have demonstrated his ability to overcome complex challenges through data-driven initiatives.

Beyond his clinical and scientific contributions, Dr. Hymes is known for his principled leadership, compassion, and sense of humor. His ability to inspire and mentor colleagues has left an indelible mark on the nephrology community. As we celebrate his retirement, we reflect on the legacy he leaves behind—a legacy of innovation, excellence, and unwavering dedication to patient care.

This Festschrift brings together the voices of colleagues and friends who have had the privilege of working with Dr. Hymes. Through their presentations, we gain insight into the profound impact he has had on the field of nephrology and the lives of those he has touched. It is our hope that this collection of tributes serves as a fitting testament to Dr. Hymes’ extraordinary career and enduring contributions.

## Leveraging the power of data in healthcare and clinical algorithms

Since the turn of the century, data has been considered the currency of health care. In 2007 Fresenius Medical Care North America (FMCNA) began the roll out of eCube Clinicals, an electronic health record (EHR) for people on dialysis. Migrating from the legacy system to a robust EHR created the opportunity to expand the company’s collection of important aspects of patient care and to store those as discrete data elements. Today the FMCNA data warehouse includes records for almost 2 million unique patients. Those records include a cornucopia of data including treatment data, medications, diagnoses, laboratory data, patient symptoms and a host of other features.

Mountains of data alone, however, provide very little value. Thoughtful interrogation of the data can lead to new insights regarding the care of patients with ESKD. Over the past 20 years, FMCNA has published many of these insights, advancing the renal community’s knowledge and leading to improvements in the care of patients with ESKD. But the true value of the data is the generation of actionable insights.


Data→Insights→Action

During his career at FMCNA, Dr. Jeff Hymes has excelled at uncovering actionable insights. Under his leadership, advanced analytics teams have utilized this massive data store to guide therapy, improve outcomes, and save lives. A few of the multitude of projects Dr. Hymes has led are highlighted in this article. The scope and scale of the FMCNA data asset is beyond comparison in the renal space. But uncovering a rare and important side effect requires leadership to direct a disciplined analysis of the data. As a member of the team who led this assessment, Dr. Hymes’ work saved lives.

### Clinical algorithms

Clinical algorithms are a sequential list of clinician decision-making steps that are designed to guide patient care. Algorithms assist with decisions in the management of complex patients while helping with resource allocation, improving efficiency of workflows, reducing clinical errors, and improving overall clinical outcomes. Physicians and health care providers use clinical algorithms to translate complex clinical data into actionable insights to enhance clinical decision making, thereby liberating clinicians to focus on patient care (1–3).

The management of ESKD patients is complex, requiring structured decision-making tools to guide healthcare providers at the point-of-care to individualize appropriate treatment plan by personalizing dialysis prescriptions and medications while monitoring complications. In January 2011, the Center for Medicare and Medicaid Services (CMS) implemented a new Prospective Payment System (PPS) for Medicare beneficiaries, known commonly as the “bundle”. The bundle includes a single payment for the dialysis treatment encompassing technical, personnel, operational, and medication expenses. The move to a bundled payment system became a catalyst to define processes focused on efficient use of resources and medications during the dialysis treatment. Jeff Hymes championed the use of clinical algorithms to manage erythropoiesis stimulating agents (ESAs) that treat anemia of chronic kidney disease (CKD) and vitamin D analogs that manage abnormalities in bone and mineral metabolism.

### The work with clinical algorithms continues

Over the course of his career, Dr. Jeff Hymes has dedicated himself to using data to uncover and deploy actionable insights. His work on ESA utilization, calcimimetics, and hepatitis C reflects a very small sample of the projects he has led. Uncovering actionable insights hidden under a mountain of data and then deploying those insights across the country requires a leader with remarkable clinical acumen and the respect of their peers. During his time at FMCNA, Jeff has filled that role admirably. Without question, his work in this capacity created benefit for FMCNA, but more importantly, it improved the care provided to the patients FMCNA serves. Dr. Jeff Hymes has paved the path for the use of clinical algorithms across FKC clinics. Initially, many nephrologists were reluctant to lose their autonomy to manage their patients individually and resisted the use of standard algorithms and protocols. The successful rollout of the Mircera algorithm for anemia management paved the way for broader adoption of clinical algorithms in FKC clinics. Over the last decade, FMCNA has gone from roughly 10 algorithms to more than 45 clinical algorithms and protocols to assist nephrologists in the management of ESKD patients on dialysis. Today, we employ algorithms to support the management of nearly all disease states in dialysis patients, including bone and mineral metabolism, fluid status and hypertension, acute kidney injury, bloodstream infections, central venous catheter reduction, peritoneal dialysis and home hemodialysis prescription, among others.

There is a project underway to create electronic algorithms utilizing no-code technology by dedicated clinical algorithm builders that provide recommendations at the point of care. These digitized electronic algorithms are built on established clinical practice guidelines and will provide real time decision support, integrate seamlessly with electronic health records, thereby improve the efficiency with which care is delivered and reduce delays in treatment. Predictive analytic tools can further help with recommending appropriate dose changes of medications and enhance the digitized algorithms to individualize their recommendations. In the next couple of years, the project’s goal is to digitize all the existing FKC clinical algorithms.

## Introduction of Mircera to Fresenius Kidney Care Clinics and development of anemia management algorithms

### Background

One of the consequences of kidney failure is the loss of the production of erythropoietin. The release of epoetin alfa on June 1, 1989, was a pivotal step in the treatment of patients with ESKD. The introduction of recombinant human erythropoietin (epoetins) revolutionized the management of anemia in CKD patients. Prior to this, anemia was managed with blood transfusions and anabolic steroids. During his storied career, Dr. Hymes has led teams to successfully leverage data to optimize the utilization of ESAs.

The early epoetins (alpha and beta) were short acting agents requiring parenteral administration thrice weekly. The short acting epoetins resulted in high variability in hemoglobin (Hg) levels due to missed doses (e.g. from a hospitalization), while increasing staff burden from frequent dosing requirements. Consequently, there was an increased desire for longer-acting ESAs (4). Many pharmaceutical companies pursued the development of compounds with a longer half-life. In the late 1990s, Darbopoietin alpha, a second-generation ESA that contained two additional N-linked carbohydrate chains to increase its half-life, became available. Darbopoietin was approved by the FDA in 2001 and was injected every two weeks.

### Peginesatide

One longer-acting ESA to achieve FDA approval was peginesatide which was approved in 2012. Peginesatide, a synthetic pegylated dimeric peptide, had no structural homology to other ESAs. It was able to stimulate erythropoiesis even in the presence of anti-erythropoietin antibodies. The medication’s approval followed two randomized controlled trials (RCTs) which found that the effectiveness of peginesatide was not inferior to epoetin for patients receiving dialysis (the EMERALD study) (5), or to darbepoetin for patients with chronic kidney disease who were not receiving dialysis (the PEARL study) (6).

Peginesatide was introduced in Fresenius Kidney Care clinics post-approval. As FMCNA began utilizing this agent, Dr. Hymes was a principal member of the team that designed a careful rollout which included the meticulous capture and timely review of hundreds of data points. The fact that FMCNA had a history of capturing such data for patients utilizing existing ESAs permitted the organization to compare outcomes at a scale that was not present in the RCTs that preceded the medication’s approval by the FDA. Thanks to the team’s close monitoring of the data, a signal was detected suggesting the new agent had a rate of serious hypersensitivity reactions, including life-threatening anaphylaxis, which was an order of magnitude higher than usual therapy. FMCNA promptly ceased the use of the agent and notified the manufacturer and the FDA who subsequently suspended marketing the agent in February of 2013 (7, 8).

### Introduction of methoxy polyethylene glycol epoetin beta in Fresenius Kidney Care clinics

With continued interest in a long-acting ESA, the continuous erythropoiesis receptor activator (CERA) containing a large pegylated polymer chain and having an extended half-life compared to Darbopoietin alpha, was of interest as it afforded administration every two weeks or monthly. Methoxy polyethylene glycol epoetin beta (Mircera), a long-acting ESA, received regulatory approval in the European Union, Canada, Japan, Australia, and received FDA approval in 2007. It entered the US market in 2014 after a prolonged patent litigation. Jeff Hymes introduced Mircera to FKC clinics in the summer of 2014 when the appetite for a long acting pegylated agent was very small after the Peginesatide withdrawal. Knowing that he needed the support of the nephrologists, Jeff wrote an extremely thoughtful white paper in December 2014, introducing Mircera to the FKC affiliated nephrologists. In this white paper, he discussed the structure and pharmacokinetics of Mircera and discussed in detail the safety and efficacy profile of the medication. He directly addressed many of the nephrologists’ concerns with long-acting ESAs, thereby gaining their confidence for the new ESA.

Mircera was introduced in a very meticulous and thoughtful way into FKC clinics by Dr. Hymes. The initial Mircera algorithm (version 1.0) focused on the conversion of Epogen and Darbopoietin to Mircera every 2 weeks. The algorithm introduced the titration of Mircera based on data gathered from phase 3 pivotal studies (9). In early 2015, a new version of the Mircera algorithm was created (version 2.0) that introduced a monthly dosing regimen as well as identified the lowest effective dose of Mircera. Subsequently, Mircera algorithm version 3.0 introduced a smaller, 30 μg starting dose of Mircera. This algorithm gained significant traction among nephrologists and was widely adopted across FKC clinics. In 2016, an analysis of the Mircera algorithm version 3.0 determined the need to reduce the number of patients with Hg above 11 g/dL, avoid patients with Hg below 10 g/dL, and increase patients within Hg range of 10 to 11 g/dL while reducing Hg variability and overall patient exposure to Mircera. A computer-based analysis by the scientists at the Renal Research Institute in New York resulted in the creation of a new Mircera algorithm (version 4.0).

This version 4.0 was built on a physiology-based mathematical models of erythropoiesis (10–12). The Mircera algorithm version 4.0 was released in a well-designed cluster randomized model so that the results could be compared with version 3.0 (**Table 1**). Additional features of the Mircera version 4.0 algorithm included directions for use in pediatric patients, recommendations to hold Mircera when Hg is greater than 11.2 g/dL and a modified titration schedule for Mircera. Later, an individualized anemia therapy assistance software was developed and put to test in an RCT in hemodialysis patients with high Hg variability. The results of this RCT, recently published by Fuertinger et al. (13), showed improved attainment of hemoglobin target while reducing variability compared with the standard of care. Personalized anemia therapy models will likely change the way we manage anemia in dialysis patients in the future.

**Table 1:** Comparison of hemoglobin outcomes between version 3.0 (V 3.0) and version 4.0 (V 4.0) of the Mircera algorithm in Fresenius Kidney Care clinics.

Algorithm Version	Hemoglobin concentration [g/dL]						
	<7	7-8	8-9	9-10	10-11	11-12	>12
V 3.0 [%]	0.11	0.87	3.29	9.81	55.08	28.56	2.29
V 4.0 [%]	0.05	0.71	3.13	11.8	58.61	23.33	2.36

The number of patients in the hemoglobin 10–11 g/dL range increased from 55.08 to 58.61%, while the percent of patients in the 11–12 g/dL range dropped from 28.56 to 23.33%. While the number of patients in the 9–10 g/dL range increased slightly, all other groups of low hemoglobin decreased.

Source: Fresenius Kidney Care data.

### Relative safety of Mircera

In June 2019, a Japanese study by Sakaguchi et al. indicated that long-acting ESA use was associated with a 13% higher rate of cardiovascular and non-cardiovascular mortality when compared to short acting ESAs during a 2-year follow up period (14). The FKC experience with Mircera was very good and did not match the results of the study. Subsequently, Jeff Hymes convened a group of clinical experts to do a deep assessment of the study. The experts concluded that the observational study by Sakaguchi et al. had significant methodological flaws including unmeasured confounding factors, missing patient data, and incomplete information on ESA exposure. Soon afterwards, Locatelli et al. (15) published the results of a multicenter, randomized, open-label non-inferiority trial (MIRCERA PASS trial) where treatment with Mircera resulted in rates of major cardiovascular events and mortality that were similar to rates with the reference ESAs, while maintaining comparable Hg levels. The authors concluded that treatment with Mircera was safe with comparable rates of major cardiovascular events, all-cause mortality, and other serious adverse events with conventional reference ESAs.

### Evaluation of Mircera in peritoneal dialysis patients

FKC clinics began using Mircera once monthly to manage anemia of CKD in PD patients beginning in 2015. The clinical experience with Mircera in PD patients was published in September 2020 by Stennett et al. (16). The study concluded that the conversion from short and medium acting ESAs to Mircera resulted in a decrease in mean ESA dose, an increase in patients on ESA dosing hold, and a change to administration of ESA by the nurse in the outpatient clinic setting, while maintaining anemia targets.

### Optimizing iron management for patients with functional iron deficiency

Many ESKD patients have functional iron deficiency that presents a suboptimal response to ESA. These patients respond well to intravenous iron therapy thereby reducing the ESA dose required to maintain stable Hg levels. There is a lack of consensus on a target ferritin level in HD patients. Consequently, there is a lack of universal recommendations regarding the serum ferritin level at which iron supplementation should be discontinued. This is complicated by the fact that ESA hyporesponders have underlying inflammation and elevated ferritin levels. The DRIVE trial found that ESA hyporesponders had a higher mortality compared to ESA normoresponders (17). They also experienced higher cardiovascular disease incidence rates and increased healthcare utilization, including hospitalization. After the PIVOTAL Study (18), Jeff Hymes helped drive more aggressive management of functional iron deficiency and to target ferritin levels between 1,000 and 1,200 μg/L in patients who have levels of transferrin saturation (TSAT) below 30%.

### Additional algorithms for management of anemia

Over the years, FKC, under the guidance of Dr. Hymes, released a Mircera Hyporesponse Algorithm that provides a comprehensive evaluation and management of ESA hyporesponsiveness. Additionally, the iron management algorithm was modified to include workup and management of functional iron deficiency. A few years ago, the Mircera algorithm version 4.0 was converted to an electronic algorithm on Chairside for hemodialysis patients with recommendations for ESA dosing provided at the point of care.

An anemia case management team monitors patients with functional iron deficiency and Mircera hyporesponsiveness and provides guidance and training to the clinicians. With the interventions by the centralized anemia case management team, the ESA dose trend has decreased significantly in the patients deemed hyporesponsive to Mircera.

## Bone and mineral metabolism

### Increased use of oral calcitriol

Intravenous vitamin D analogs used to be the first-line therapy for management of secondary hyperparathyroidism in US hemodialysis patients. This practice was probably driven by studies from the US that indicated that these drugs were associated with fewer episodes of hypercalcemia and hyperphosphatemia while achieving bone and mineral metabolism clinical outcome targets. Subsequent small studies found no difference in PTH management or risk of hypercalcemia with oral calcitriol (1,25-dihydroxyvitamin D3) vs. intravenous vitamin D analogs.

Under Jeff’s guidance, a retrospective cohort study of adult patients treated within FKC clinics by Thadhani et al. conducted in FKC clinics showed that when 2,280 patients who switched to oral calcitriol were matched to 2,280 patients on intravenous vitamin D analogs, the aggregate of all mineral and bone laboratory parameters in range was largely similar between groups and that calcium and phosphorus in the oral calcitriol group was lower after the switch (19). The increased use of oral calcitriol in the US is largely attributed to the results of this study and Jeff’s leadership in promoting the use of oral calcitriol in FKC clinics.

### Calcimimetics

The treatment of chronic kidney disease-mineral and bone disorder (CKD-MBD) is one of the most complex tasks faced by the ESRD patient’s care team. In 2004, this complexity increased as a new class of medications became available with the release of cinacalcet, a calcimimetic drug that lowers the level of parathyroid hormone (PTH). This oral calcimimetic was rapidly adopted, and in 2017 a second calcimimetic became available as an injectable agent (etelcalcetide). This launched the first encounter with the ESKD PPS Transitional Drug Add-on Payment Adjustment (TDAPA) process for the organization. In the face of uncertainty, Dr. Hymes led a team of individuals who analyzed mountains of longitudinal data to create effective algorithms for the utilization of calcimimetics in a fiscally responsible manner. Absent this thoughtful, data driven approach, calcimimetic utilization, both during and after the TDAPA period would have been chaotic at best.

Given the frequent gastrointestinal side effects of cinacalcet and the resulting poor adherence to daily dosing, Dr. Hymes initiated research into thrice-weekly oral administration. Novel physiology-based mathematical models demonstrated that controlled thrice-weekly dosing during hemodialysis provided parathyroid hormone (PTH) control that was non-inferior to daily dosing (20, 21). Following these findings, thrice-weekly cinacalcet dosing was widely adopted across FKC clinics, with PTH outcomes aligning with model prediction (22).

## Infectious diseases

### Antibiotic stewardship

Antibiotic stewardship is an approach aimed at optimizing the use of antibiotics to combat antibiotic resistance and improve patient outcomes. This involves prescribing antibiotics only when necessary, selecting the appropriate antibiotic, and determining the correct dose and duration of treatment. Antibiotic stewardship can reduce the misuse and overuse of antibiotics, contributing to the rise of antibiotic-resistant bacteria. Antibiotic Stewardship programs are essential in preserving the effectiveness of current antibiotics (23).

One effective tool within antibiotic stewardship is the use of treatment algorithms. Algorithms provide a standardized, evidence-based approach to assist clinicians with therapy selection. By following a treatment algorithm, healthcare providers can consistently choose the most appropriate antibiotic therapy based on the specific infection, patient factors, and local resistance patterns. Treatment algorithms reduce variability in prescribing practices and promote the use of first-line antibiotics. These algorithms facilitate rapid decision-making, ensuring timely treatment and minimizing unnecessary antibiotic exposure.

Reducing exposure to inappropriate antibiotics also helps to decrease the likelihood of bacterial resistance. For example, while aminoglycosides are effective in specific situations, they are not always the best choice. By carefully selecting antibiotics with a less narrow therapeutic index and with an appropriately focused spectrum of activity, healthcare providers can preserve aminoglycosides for cases where they are the most appropriate option (24).

Vancomycin trough monitoring helps optimize dosing, ensuring patients receive the minimum effective dose and avoiding excessive levels that can lead to toxicity. By maintaining appropriate trough levels, healthcare providers can individualize vancomycin therapy, which improves treatment outcomes and reduces the risk of resistance. Inappropriate dosing can lead to subtherapeutic levels, contributing to treatment failure and encouraging bacterial resistance (25).

Dr. Hymes has been instrumental in advancing antibiotic stewardship in dialysis settings, focusing on optimizing antimicrobial use through evidence-based algorithms and monitoring. Recognizing the risks associated with prolonged antibiotic exposure in dialysis patients, particularly nephrotoxic agents like aminoglycosides and vancomycin, he has championed initiatives to minimize unnecessary antibiotic use. His efforts include implementing treatment protocols that guide clinicians in selecting appropriate antibiotics, dosing regimens, and treatment durations tailored to individual patient needs. By reducing exposure to aminoglycosides and optimizing the dose and duration of vancomycin, Dr. Hymes’ approach helps mitigate the risk of antimicrobial resistance. Through continuous monitoring and adherence to best practices, his work has significantly enhanced the safe and effective use of antibiotics in this vulnerable patient population.

### Hepatitis C

Since its identification in 1989, hepatitis C has been recognized as a devastating condition in those with ESKD. Unchecked, hepatitis C can lead to hepatic fibrosis, cirrhosis, and hepatocellular carcinoma. Early treatment options were largely ineffective and carried the risk of multiple side effects in patients with ESKD. This changed with the advent of a class of medications referred to as direct acting antiviral (DAA) agents. DAA medications have revolutionized HCV treatment in dialysis patients and offer highly effective and well-tolerated options with significantly improved cure rates. The early utilization of DAAs held promise for hepatitis C patients, but unfortunately the new agents were not available to patients with ESKD due to their renal metabolism and excretion. That all changed in 2016 with the release of Zepatier™ (elbasvir/grazoprevir), an oral DAA approved for use in ESKD patients with a demonstrated sustained viral response rate exceeding 95% after a 12-week course of treatment.

Despite the presence of a kidney friendly DAA agent, a number of barriers stood between the medical office and the successful deployment of this curative therapy including controversy related to screening, the need to ensure the daily regimen was adhered to, and access to hepatologists among many others. Given the complexity surrounding this valuable opportunity, Dr. Hymes once again led a team that leveraged data from the FMCNA data warehouse to overcome these challenges and launched a major initiative to diagnose, treat and eradicate hepatitis C in FKC facilities. Jeff promoted the management of hepatitis C in FKC dialysis patients and set up a process for the medications to be prescribed and managed by nephrologists under the guidance of a hepatologist. He led the charge in introducing safe and effective treatment for hepatitis C in patients with advanced kidney disease, leading to eradication of this infection for nearly all treated patients.

### Vaccinations

Realizing that ESRD patients are hyporesponsive to hepatitis B vaccines, Dr. Hymes helped introduce a novel adjuvanted hepatitis B vaccine that saw a higher seroconversion rate, a lower revaccination rate, and sustained response compared to previously available HBV vaccines. Through his leadership and encouragement, FKC influenza vaccination rate remains nearly 90% for all eligible patients. Standardizing pneumococcal vaccination can be very complex but is made more manageable with algorithms to ensure patients are current on vaccinations consistent with guideline recommendations.

### Prevention of blood stream infections

The rate of bloodstream infections (BSIs) is disproportionately high in HD patients with central venous catheters (CVCs) versus those with permanent accesses, contributing to poorer outcomes, such as increased rates of death and hospitalizations. Dr. Hymes addressed this problem on multiple levels. He pioneered the use of chlorhexidine embedded catheter caps in dialysis patients with CVC for infection prevention and led a cluster-randomized trial that showed that use of such caps, when compared with standard CVC caps, significantly lowers rates of catheter-related BSI and hospital admissions for BSI in HD patients using CVCs (26). Moreover, standardizing infection prevention, such as “scrub the hub” protocol, across all FKC dialysis facilities reduced BSI below the National Healthcare Safety Network (NHSN) Standardized Infection Ratio.

### The COVID-19 pandemic

The COVID-19 pandemic represented a watershed moment for the field of kidney care with all of the vulnerabilities of the population of patients served impacted by the viral infection or the disruption of usual care in health systems across the world. Dr. Hymes was a leader in aggressive use of personal protective equipment (PPE) for both staff and patients and the careful consideration of the use of isolation clinics for patients that either suffered from COVID or had high exposure potential. He was the calming force in the proactive vaccination efforts for our patients (27) and the early treatment when patients presented with early signs and symptoms. He helped introduce predictive capabilities (28) by analyzing temperature and cough screens which predicted spikes in COVID cases and promoted the most aggressive hygiene measures to allow for continued care of a population of patients that needed their life sustaining dialysis therapy.

## Physician leadership and mentorship

Physician leadership is more critical today than at any time in the recent past as a result of the increasing complexity of healthcare systems and the rapid pace of medical advancements. The shift towards value-based care and the growing demand for improved patient outcomes require leaders for whom not only healthcare administration but also clinical practice are fully ingrained. Physicians, with their firsthand experience in patient care, are uniquely positioned to lead these efforts. They can bridge the gap between administrative goals and clinical realities, ensuring that organizational strategies align with the best interests of patients. As health care has become more focused on outcomes, physician leaders are an essential in translating clinical expertise into effective policies and practices. Nowhere in Medicine is that more true than in dialysis. Dr. Hymes was on the front lines for dialysis for several decades advocating for, creating, and implementing quality improvement within dialysis organizations in anemia management, bone mineral metabolism, fluid management among many, resulting in improvements during his tenure at FKC in CMS 5-Star ratings for clinics and improved clinical quality scores for patients within FKC. This required operational savvy in addition to medical knowledge to navigate the clinical organization at scale, in recent years ~2,700 dialysis units and ~50,000 employees - mostly involved in direct patient care. Dr. Hymes managed relationships with medical directors for these clinics with approximately 45% of those clinics representing joint venture partnerships with nephrologists and nephrology practices. He provided mentorship of up-and-coming leadership within FKC, such as Drs. Dinesh Chatoth, Milind Nikam, Adrian Ginsburg, and Chance Mysayphonh. As a member on the Renal Physicians Association (RPA) board of directors he balanced his FKC corporate role while acting as a strong advocate for individual nephrologists in the United States. Jeff provided testimony to the US federal government regarding delivery of dialysis care through dialysis organizations.

The COVID-19 pandemic provided an extreme test of leadership, and we saw first-hand Dr. Hymes rise and meet the medical challenge. Physician leadership played a pivotal role in managing the pandemic response at large, in close working partnership with Kathleen Belmonte, RN, chief nursing officer at FKC, helping to guide dialysis organizations through unprecedented challenges such as shortages in personal protective equipment (masks, gloves, gowns and more), in medical supplies, and in staffing. It included evolving treatment protocols (sometimes changing daily) and managing overwhelming patient volumes in settings like isolation clinics, critical care units, and multiple hospital locations. The ability to combine clinical knowledge with organizational leadership allowed Dr. Hymes to help not just FKC but support other dialysis organizations through regular virtual meetings with the Chief Medical Officers of other dialysis organizations adapt rapidly while maintaining high standards of care.

In a world increasingly vulnerable to public health crises, physician leadership is crucial in ensuring that healthcare organizations are prepared, resilient, and capable of responding effectively to future challenges. Events beyond the COVID-19 pandemic have highlighted the critical role of physician leadership during crises. Dr. Hymes led FKC’s disaster response efforts (29), which included coordinated actions during hurricanes, earthquake relief efforts (e.g., in Turkey), the military conflict in Ukraine (30), and responses to terrorism and armed conflict, such as the October 2023 Hamas attack on Israel.

The rise of digital health technologies, such as telemedicine, artificial intelligence (31, 32), and precision medicine also elevates the importance of physician leadership. These technologies are transforming the way healthcare is delivered and were heavily on display in the pandemic, but they require leaders with deep understanding of both medicine and technology to ensure that innovations are implemented in ways that enhance care quality and patient safety. Dr. Hymes is a physician leader who has helped guide these technological advancements, ensuring that they are adopted in a manner that complements clinical workflows and improves patient outcomes. As the healthcare industry continues to evolve at its rapid pace, the role of physician leadership in steering organizations through these changes has never been more vital.

For us, his colleagues, Dr. Hymes is someone with whom we have had the opportunity to work closely for the last 12 or more years providing us with a view of leadership that confirms that “character counts.”

Character, exemplified by the eight traits outlined below, allowed for skillful navigation to patient and business benefit, through daily routines and once-in-a-century-pandemic challenges, and everything in between.

Principled: strong sense of what’s right and doing the right thing

Exacting - Demanding: expectations of those around him; raising the bar

Personable: good interpersonal skills and cultural sensitivity

Loyal: to family; to patients; to colleagues and staff; to the company

Evidence based: presentations known for focus on quality and efforts to improve

Compassionate: focused on care and service to patients and families

Funny: great source of funny stories well told

Able to compromise - especially if given the opportunity to “sleep on it”

Principles consistently were on display with Dr. Hymes’ strong sense of what’s right and doing the right thing. In pressured settings with limited time and incomplete information, these guided his recommendations and decisions.

He was exacting. High expectations of those around him helped raise the bar (and the performance) of all, knowing that he was most demanding of himself.

Jeff is also very personable with good interpersonal skills and cultural sensitivity that enabled him to work well with others - invaluable skills in large organizations where communication, collaboration, and cooperation are central to success.

Jeff’s words and actions showed his loyalty to family, to patients, to colleagues and staff, and to the organization. These were woven seamlessly into the fabric of daily work and life to achieve the best balance in the moment.

Dr. Hymes was evidence based in his approach to all things work. His presentations were known for focusing on quality of care and efforts to improve whatever issue was at hand.

This was done with a deliberate sense of compassion focused on care and service to patients and families while recognizing the need to be attentive to staff and to business leaders and business realities/goals.

In no small measure, the fact that Jeff knows how to be funny, and the right time to employ a sense of humor, helped a great deal. Jeff is a source, a great source, of funny stories well told.

And finally, Dr. Hymes was able to compromise - especially if given the opportunity to “sleep on it.”

The ability to truly listen and incorporate additional thinking in decision making without abandoning principles and undermining best practices even as such “best practices” are being developed “real-time” as they were during the public health emergency declaration period of COVID-19, is a key differentiator of a leader vs. an administrator or a manager.

Jeff is one of our sources of inspiration, one of our role models, and our friend.

## Data Availability

The original contributions presented in the study are included in the article/Supplementary Material. Further inquiries can be directed to the corresponding author.
